# Supplementary material for: Fabry–Pérot resonances and a crossover to the quantum Hall regime in ballistic graphene quantum point contacts
Source: Sci Rep. 2019 Feb 28;9:3031. doi: 10.1038/s41598-019-39909-5 (PMC6395604; doi:10.1038/s41598-019-39909-5)
Supplement: Supplementary file 1 — Supplementary Information [file 41598_2019_39909_MOESM1_ESM.pdf]

Supplementary Information

Fabry–Pérot resonances and a crossover to the quantum Hall regime in ballistic graphene quantum point contacts

Nurul Fariha Ahmad[1,2], Katsuyoshi Komatsu[1], Takuya Iwasaki[3], Kenji Watanabe[4], Takashi Taniguchi[4], Hiroshi Mizuta[5,6], Yutaka Wakayama[1], Abdul Manaf Hashim[2], Yoshifumi Morita[7], Satoshi Moriyama[1], Shu Nakaharai[1†]

[1]International Center for Materials Nanoarchitectonics (WPI-MANA), National Institute for Materials Science (NIMS), Tsukuba, Ibaraki 305-0044, Japan.

[2]Malaysia-Japan International Institute of Technology, Universiti Teknologi Malaysia, Jalan Sultan Yahya Petra, 54100 Kuala Lumpur, Malaysia.

[3]International Center for Young Scientists (ICYS), National Institute for Materials Science (NIMS), Tsukuba, Ibaraki 305-0044, Japan.

[4]Research Center for Functional Materials, NIMS, Tsukuba, Ibaraki 305-0044, Japan.

[5]School of Material Science, Japan Advanced Institute of Science and Technology, Nomi, Ishikawa 923-1211, Japan.

[6]Hitachi Cambridge Laboratory, Hitachi Europe Ltd., J. J. Thomson Avenue, Cambridge, United Kingdom.

[7]Faculty of Engineering, Gunma University, Kiryu, Gunma 376-8515, Japan.

† Corresponding author. Email: NAKAHARAI.Shu@nims.go.jp

This PDF file includes:

・FIG. S1. Electrostatic-potential simulation of our QPC device
・FIG. S2. Simulation of the Fabry–Pérot resonances
・note S1. Modeling of the Electrostatic Potential
・note S2. Simulation of the Fabry–Pérot resonances

S1. Modeling of the Electrostatic Potential

We performed a simulation of the electrostatic-potential based on the method of ref. [16, 24], which lead to our picture of cavity formation by the top gate. Figure S1(a) shows the schematic of our device for the simulation. We employed parameters for the model to simulate the experimental devices. The model consisted of a quantum point contact (QPC) geometry with a gap of 60 nm covered by a vacuum box. The simulation was aided by COMSOL Multiphysics (COMSOL Inc.), a modelling and calculating software based on the finite element method. During the simulation, we applied voltage to the split top gates and grounded the graphene layer, obtained the electric field (E_z) in the z-direction at the surface of graphene in a self-consistent manner and calculated the Fermi energy (E) of graphene using the formula, $E = \mathrm{sgn}(-E_z) \times \hbar v_F \sqrt{\pi |E_z| \varepsilon_0 \varepsilon_{hBN} / e}$, where $\hbar$ is the reduced Planck constant, v_F ~10^6 m/s is the Fermi velocity of graphene, e is the elementary charge and ε_0 and ε_{hBN} = 3.9 are the vacuum permittivity and the dielectric constant of hexagonal boron nitride, respectively.

Figure S1(b) shows the electrostatic-potential mapping at the surface of the graphene layer where the origin (0,0) corresponds to the centre of the QPC. Figure S1(c) shows the energy profile along with the x-direction at y = 200 nm (across the split top-gate). The energy under the top gate increased as the top-gate voltage was increased, indicating the formation of the cavity for Fabry–Pérot resonances.

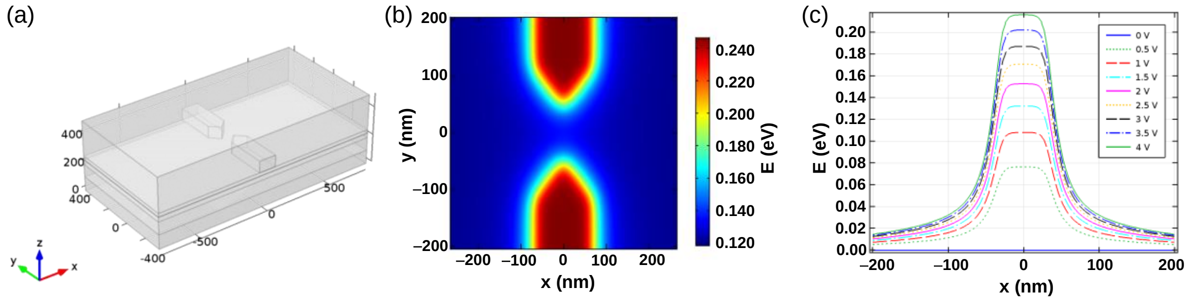

FIG. S1: Electrostatic-potential simulation of our QPC device. (a) Schematic illustration of the QPC device model (length unit: nm). (b) Mapping of the Fermi energy of graphene at the top surface for application of 4 V top-gate voltage. (c) Energy profile along the x-direction across the region under the top-gate (y = 200 nm) for various top-gate voltages.

S2. Simulation of the Fabry–Pérot resonances

To substantiate our picture and fix characteristic energy scales, we performed a simulation of the transmission in our device through QPC. We applied WKB analysis based on the cavity model established in [15, 16]. Our focus was on clear FP resonances around $|V_{TG}|$>~1V (Figs.

3 and 4 in our manuscript) where the half-a-period('π') phase shift takes place under a finite magnetic field, i.e. a signature of Klein tunnelling. A typical solution for the WKB analysis is given in Fig. S2 in a normalised unit and discussed below, while the oscillating part was focused on as in [16]. Results in S1 confirmed the basic characteristic scales [15]. Applying a typical value of an order 1 eV/μm^2 for the curvature of the potential (=a), we found a characteristic energy unit scale $(a v_F^2 \hbar^2)^{1/3}$(=ε_*) of ~10 meV. Considering the conversion factor from the top-gate voltage ~1/25, the basic energy scale for FP resonance was consistent (Fig. S2). Furthermore, we determined a typical unit scale for the magnetic field $(\Phi_0/2\pi(\hbar v_F/\varepsilon_*)^2)$ of ~0.1 T (Φ_0: unit magnetic flux), which is compatible with the magnetic field for the π-shift. Although this model was simplified, it gave a basic unit for the energy/magnetic field scales of the experimental results, combined with the data in S1. On the other hand, the resonance amplitude should be suppressed due to e.g. roughness in the cavity surface and finite temperature effects. For a discussion of the fine structures of the quantum interference pattern, more details of the QPC structure and its electrostatic profile will be provided in a separate paper.

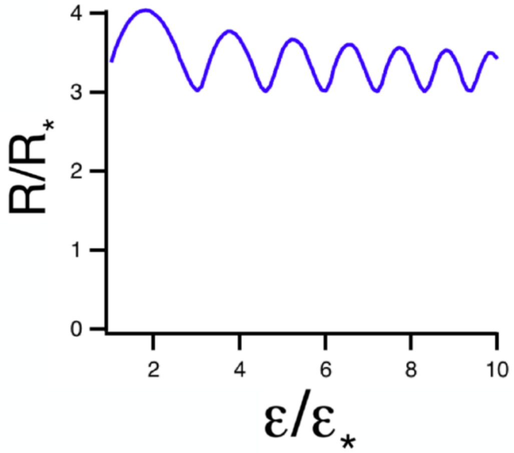

FIG. S2: Simulation of the Fabry–Pérot resonances. Typical resistance oscillation (in units of quantum resistance/scaled by a normalised cavity width) is shown as a function of energy at B = 0 T. Our focus is on the oscillatory behaviour and the offset is shifted for clarity. The unit scales are discussed above.
